# Correction: A novel biomass-based support (Starbon) for TiO_2_ hybrid photocatalysts: a versatile green tool for water purification

**DOI:** 10.1039/c8ra90052c

**Published:** 2018-06-19

**Authors:** Juan Carlos Colmenares, Paweł Lisowski, Dariusz Łomot

**Affiliations:** Institute of Physical Chemistry PAS Kasprzaka 44/52 01-224 Warsaw Poland jcarloscolmenares@ichf.edu.pl

## Abstract

Correction for ‘A novel biomass-based support (Starbon) for TiO_2_ hybrid photocatalysts: a versatile green tool for water purification’ by Juan Carlos Colmenares *et al.*, *RSC Adv.*, 2013, **3**, 20186–20192.

The authors regret that the Experimental section (2.2 Preparation of hybrid TiO_2_-based carbonaceous materials) of the original article requires a small correction. There was apparently some minor arithmetic error involved in the calculation of the nominal mass percentage of TiO_2_ which was deposited on carbonaceous carbon materials. The nominal mass percentage of TiO_2_ deposited on carbonaceous carbon materials was calculated incorrectly (should be 20.9 wt% TiO_2_ instead of 25 wt% TiO_2_). It should be recognized that this error does not affect any discussion and conclusions reported in this publication. However, this correction is necessary to determine the nominal mass percentage of TiO_2_ which was deposited on carbon materials and improve the quality of this scientific publication.

The Royal Society of Chemistry apologises for these errors and any consequent inconvenience to authors and readers.

## Supplementary Material

